# Efficacy of umeclidinium/vilanterol versus umeclidinium and salmeterol monotherapies in symptomatic patients with COPD not receiving inhaled corticosteroids: the EMAX randomised trial

**DOI:** 10.1186/s12931-019-1193-9

**Published:** 2019-10-30

**Authors:** François Maltais, Leif Bjermer, Edward M. Kerwin, Paul W. Jones, Michael L. Watkins, Lee Tombs, Ian P. Naya, Isabelle H. Boucot, David A. Lipson, Chris Compton, Mitra Vahdati-Bolouri, Claus F. Vogelmeier

**Affiliations:** 1 Centre de Pneumologie, Institut universitaire de cardiologie et de pneumologie de Québec, Université Laval, Québec, Canada; 2 Respiratory Medicine and Allergology, Lund University, Lund, Sweden; 3 Clinical Research Institute of Southern Oregon, Medford, OR USA; 4 Global Specialty & Primary Care, GSK, Brentford, Middlesex, UK; 5 Respiratory Research and Development, GSK, Research Triangle Park, NC USA; 6 Precise Approach Ltd, contingent worker on assignment at GSK, Stockley Park West, Uxbridge, Middlesex, UK; 7 Respiratory Research and Development, GSK, Collegeville, PA USA; 8 Perelman School of Medicine, University of Pennsylvania, Philadelphia, PA USA; 9 Respiratory Discovery Medicine, Respiratory Research and Development, GSK, Stevenage, Hertfordshire, UK; 10 Department of Medicine, Pulmonary and Critical Care Medicine, University Medical Center Giessen and Marburg, Philipps-Universität Marburg, Germany, Member of the German Center for Lung Research (DZL), Marburg, Germany

**Keywords:** COPD, Lung function, Dyspnoea, Clinically important deterioration, Bronchodilator therapy

## Abstract

**Background:**

Prospective evidence is lacking regarding incremental benefits of long-acting dual- versus mono-bronchodilation in improving symptoms and preventing short-term disease worsening/treatment failure in low exacerbation risk patients with chronic obstructive pulmonary disease (COPD) not receiving inhaled corticosteroids.

**Methods:**

The 24-week, double-blind, double-dummy, parallel-group Early MAXimisation of bronchodilation for improving COPD stability (EMAX) trial randomised patients at low exacerbation risk not receiving inhaled corticosteroids, to umeclidinium/vilanterol 62.5/25 μg once-daily, umeclidinium 62.5 μg once-daily or salmeterol 50 μg twice-daily. The primary endpoint was trough forced expiratory volume in 1 s (FEV_1_) at Week 24. The study was also powered for the secondary endpoint of Transition Dyspnoea Index at Week 24. Other efficacy assessments included spirometry, symptoms, heath status and short-term disease worsening measured by the composite endpoint of clinically important deterioration using three definitions.

**Results:**

Change from baseline in trough FEV_1_ at Week 24 was 66 mL (95% confidence interval [CI]: 43, 89) and 141 mL (95% CI: 118, 164) greater with umeclidinium/vilanterol versus umeclidinium and salmeterol, respectively (both *p* < 0.001). Umeclidinium/vilanterol demonstrated consistent improvements in Transition Dyspnoea Index versus both monotherapies at Week 24 (vs umeclidinium: 0.37 [95% CI: 0.06, 0.68], *p* = 0.018; vs salmeterol: 0.45 [95% CI: 0.15, 0.76], *p* = 0.004) and all other symptom measures at all time points. Regardless of the clinically important deterioration definition considered, umeclidinium/vilanterol significantly reduced the risk of a first clinically important deterioration compared with umeclidinium (by 16–25% [*p* < 0.01]) and salmeterol (by 26–41% [*p* < 0.001]). Safety profiles were similar between treatments.

**Conclusions:**

Umeclidinium/vilanterol consistently provides early and sustained improvements in lung function and symptoms and reduces the risk of deterioration/treatment failure versus umeclidinium or salmeterol in symptomatic patients with low exacerbation risk not receiving inhaled corticosteroids. These findings suggest a potential for early use of dual bronchodilators to help optimise therapy in this patient group.

## Background

Long-acting bronchodilators, including long-acting muscarinic antagonists (LAMAs) and long-acting β_2_-agonists (LABAs), form the basis of maintenance therapy in chronic obstructive pulmonary disease (COPD) [1]. The recommended first-line treatment for patients with symptomatic COPD at low exacerbation risk is LAMA or LABA monotherapy. LAMA/LABA dual therapy is also considered appropriate initial therapy in patients who experience severe breathlessness; however, based on current evidence, a stepwise approach is generally preferred [1].

LAMA/LABA therapy is more effective than LAMA or LABA monotherapy for improving lung function in patients with COPD; however, variability exists across studies in the reported magnitude of improvements of symptoms and health status with dual bronchodilation [2–8]. Until now, trials comparing dual- versus mono-bronchodilator therapy have generally included large proportions of patients with predominantly low exacerbation risk, but who were nevertheless using concurrent inhaled corticosteroids (ICS) [2, 5, 9, 10]. Concurrent use or recent withdrawal of ICS may limit the generalisability of the results of such bronchodilator studies and confound the results regarding the incremental benefits of LAMA/LABAs compared with mono-bronchodilator therapies [11, 12]. A recent integrated analysis of 23 randomised controlled trials (RCTs; *n* = 23,213) evaluating the effect of LAMA/LABAs versus LAMAs, LABAs or placebo, on lung function, symptoms and exacerbation rates reported that 54% of enrolled patients were using concurrent ICS [4]. In the only 24-week Phase III RCT comparing the LAMA/LABA umeclidinium/vilanterol (UMEC/VI) versus UMEC and VI monotherapy, half of the patients continued using concurrent ICS [7]. That study demonstrated improvements in lung function and rescue medication use with UMEC/VI versus both monotherapies but did not demonstrate any incremental improvements in self-reported breathlessness or other patient-reported outcomes (PROs) [7]. Patients not receiving ICS treatment are an important and prevalent category of patients and usually reflect earlier stages of COPD. Consequently, further trials of UMEC/VI versus mono-bronchodilator therapy in symptomatic patients not receiving concurrent ICS are warranted to prospectively assess treatment optimisation in this patient population.

A composite endpoint of clinically important deterioration (CID) was recently developed to assess short-term disease worsening across multiple dimensions. The CID assesses deterioration from baseline in individual patients in terms of forced expiratory volume in 1 s (FEV_1_), and/or a PRO, and/or the occurrence of a moderate or severe COPD exacerbation [8, 13–16]. Assessing both symptom improvement and risk of short-term disease deterioration (i.e. treatment failure) is important to fully quantify the adequacy of maintenance therapy in individual patients, as since many symptomatic patients fail to achieve clinically relevant improvements or achieve relative disease stability, and instead experience deterioration of their disease [14]. To date, CID has only been evaluated retrospectively in the comparison of dual-versus mono-bronchodilators [8, 13–16].

To better understand the role of dual bronchodilation in symptomatic low exacerbation risk patients, we conducted the large prospective trial, Early MAXimisation of bronchodilation for improving COPD stability (EMAX), which monitored improvements in spirometry, a range of PROs and CID, with three different bronchodilators: UMEC/VI, UMEC and salmeterol.

## Methods

### Study design

This 24-week, multicentre, randomised, double-blind, double-dummy, 3-arm, parallel-group trial (NCT03034915; GSK study: 201749) was conducted between June 2017 and June 2018 in 213 centres in Germany, USA, Argentina, Sweden, Canada, Italy, South Africa, Netherlands, Spain, Australia, France, and Mexico. Patients were randomised 1:1:1 to once-daily fixed-dose combination UMEC/VI (62.5/25 μg) via the ELLIPTA inhaler and twice-daily placebo via the DISKUS inhaler, once-daily UMEC (62.5 μg) via ELLIPTA inhaler and twice-daily placebo via DISKUS, or twice-daily salmeterol (50 μg) via DISKUS and once-daily placebo via ELLIPTA inhaler (Additional file 6: Figure S1). Salmeterol was selected as a comparator as no once-daily LABAs were approved at standard doses in all countries participating in the study; its use also allowed the LABA treatment to be easily blinded compared with the alternative twice-daily LABA, formoterol. UMEC was selected as it is a component of the dual bronchodilator and it has also demonstrated superior lung function benefits compared with tiotropium [17].

This study was performed according to the Declaration of Helsinki and received appropriate ethical approval. All patients provided written informed consent via a form signed at either the Pre-screening or Screening visit.

### Patients

Eligible patients were ≥ 40 years of age, current/former smokers (≥10 pack-years smoking history), with a COPD diagnosis (American Thoracic Society/European Respiratory Society definition), pre- and post-salbutamol FEV_1_/forced vital capacity (FVC) ratio < 0.7, post-salbutamol FEV_1_ of ≥30–≤80% predicted (Global Initiative for Chronic Obstructive Lung Disease [GOLD] stage 2/3), COPD Assessment Test (CAT) score ≥ 10, with ≤1 moderate exacerbation and no severe exacerbations in the previous year. Before screening and during the 4-week run-in period, bronchodilator maintenance therapy was limited to a LAMA or LABA. All patients were required to be ICS and ICS/LABA free for ≥6 weeks and LAMA/LABA free for ≥2 weeks prior to run-in. As-needed salbutamol was allowed throughout all study phases.

### Randomisation and masking

Eligible patients were stratified by country and long-acting bronchodilator use during run-in and were centrally randomised within each country using an interactive web response system (Registration and Medication Ordering System NG). All treatments and/or matched placebo in this double-blind, double-dummy trial were delivered via identical inhalation devices so that treatment was masked to patients, investigators and data analysts.

### Procedures

Clinic visits occurred at Pre**-**screening/Screening, Randomisation (Day 1), and after 4, 12, and 24 weeks of treatment (Additional file 6: Figure S1). At the Pre-screening visit, demographic and concomitant medication information were collected, and COPD exacerbation history was assessed. Assessment of symptom-reported outcomes at clinic visits were conducted in the following order and before other study assessments: self-administered computerised-Baseline Dyspnoea Index (SAC-BDI; at randomisation visit), self-administered computerised-Transition Dyspnoea Index (SAC-TDI; post randomisation), and global assessment of disease severity. Health status was assessed at clinic visits after the symptom-reported outcomes using St George’s Respiratory Questionnaire (SGRQ), and CAT. Daily symptoms were evaluated using rescue salbutamol use and the Evaluating Respiratory Symptoms-COPD (E-RS), which were both captured via an electronic diary (e-diary) (Additional file 1: Table S1). Exacerbations were recorded by the physician at the Pre-screening visit and at every subsequent visit until completion of follow-up contact. Exacerbations requiring treatment with oral corticosteroids and/or antibiotics were categorised as moderate, and those requiring hospitalisation or an emergency room visit as severe. CID was prospectively analysed as a composite endpoint of time to first deterioration from baseline in trough FEV_1_, SGRQ total score, CAT score, and SAC-TDI focal score in addition to the occurrence of a moderate/severe exacerbation.

### Outcomes

The primary endpoint was change from baseline in trough FEV_1_ at Week 24. Trough FEV_1_ at Week 24 was defined as the mean of the FEV_1_ values obtained 23 and 24 h after dosing on the previous day (Day 167) as recorded in the e-diary. Additional spirometry assessments included trough FEV_1_, FVC, and inspiratory capacity (IC) over 24 weeks. Patient-reported symptom-based assessments included SAC-TDI for breathlessness, global assessment of disease severity, daily rescue salbutamol use, and E-RS respiratory symptoms total score. Health status assessments included SGRQ total score and CAT score. Response rates in individual patients were defined as ≥1-unit improvement from baseline in SAC-TDI score [18], ≥2-point reduction from baseline in E-RS total score [19], ≥4-point reduction from baseline in SGRQ total score [20], and ≥ 2-unit improvement from baseline in CAT score [21].

Time to first moderate or severe exacerbation was also assessed. Risk of a first CID was assessed in individual patients according to three composite definitions: A) a first moderate or severe exacerbation, and/or a trough FEV_1_ decrease from baseline of ≥100 mL, and/or a deterioration in health status using SGRQ (≥4 units from baseline); B) as per the first definition with a CAT deterioration (≥2 units from baseline) replacing a SGRQ deterioration; C) a FEV_1_-free CID definition including a first moderate or severe exacerbation, and/or a SGRQ deterioration, and/or a CAT deterioration, and/or a TDI deterioration (≥1 unit decrease from baseline). Safety outcomes included incidence of adverse events (AEs) and serious AEs (SAEs).

### Statistical analysis

This study was powered to detect differences in the primary endpoint and in SAC-TDI at Week 24. The primary treatment comparison for the primary endpoint was UMEC/VI versus UMEC. If the primary comparison was significant, this allowed inference of other treatment comparisons. The sample size calculation used a two-sided 5% significance level and an estimate of between patient standard deviation (SD) for TDI of 2.94 units [7]. Based on these data, 727 evaluable patients per treatment arm were required to provide 90% power to detect a statistically significant difference in TDI if the true difference was 0.5 units (half the minimal clinically important difference) [18], between UMEC/VI and UMEC. With this number of evaluable patients per arm, the study would have > 99% power assuming a true treatment difference of 80 mL between UMEC/VI and UMEC for trough FEV_1_ at 24 weeks at the two-sided 5% significance level. This calculation used a trough FEV_1_ SD of 240 mL, based on prior results for trials comparing dual- versus mono-bronchodilators [2, 7]. The intent-to-treat (ITT) population included all randomised patients who received ≥1 dose of study treatment. The primary analysis was a mixed model repeated measures analysis based on a two-sided hypothesis testing approach. Least squares (LS) mean and LS mean change from baseline analyses with estimated treatment differences and 95% confidence intervals (CIs) are presented. Responder analyses with corresponding odds ratios (OR) and 95% CIs were performed using a generalised linear mixed model. Time to first exacerbation hazard ratios (HR) and 95% CIs were based on Cox proportional hazards model with covariates of treatment, stratum (number of bronchodilators per day during run-in), and geographical region. Time to first CID HRs and 95% CIs were based on a Cox proportional hazards model with covariates of treatment, stratum (number of bronchodilators per day during run-in), geographical region, trough FEV_1_ at baseline, and SGRQ score at baseline. Safety endpoints were analysed descriptively. All analyses presented were pre-planned with the exception of time to study treatment withdrawal, which was post hoc.

This study was funded by GlaxoSmithKline (GSK study number: 201749 [NCT03034915]). GSK-affiliated authors had a role in study design, data analysis, data interpretation, and writing of the report and GSK funded the article processing charges and open access fee.

## Results

**Fig. 1:**
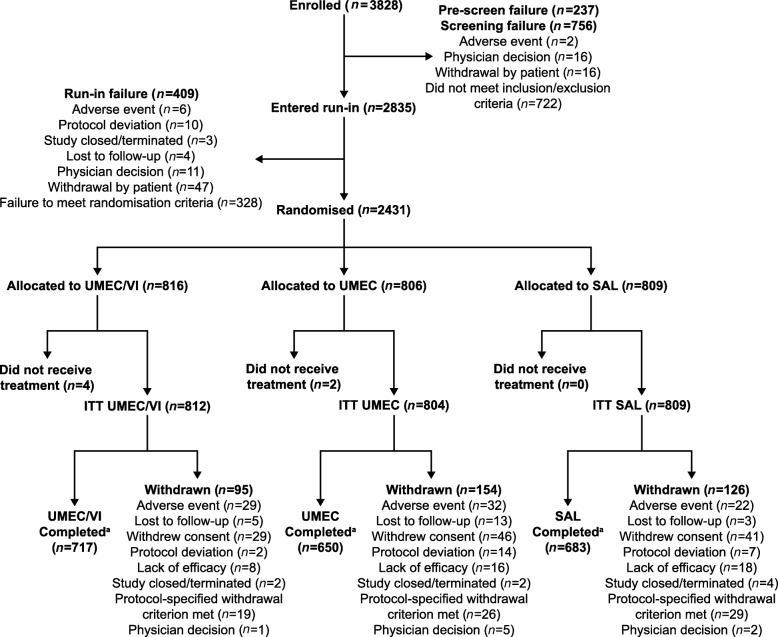
Patient disposition. ^a^Patients were considered to have completed the study if they received study treatment at Week 24 and completed the follow-up contact at Week 25 (±3 days). *ITT*, intent-to-treat; *UMEC*, umeclidinium; *VI*, vilanterol

Of 3828 patients screened, 2431 were randomly assigned to treatment; 6 patients did not receive study treatment and were excluded from the ITT population (Fig. 1). The most frequent reason for screening failure was not meeting the COPD severity inclusion criteria.

The ITT population comprised 2425 patients across the UMEC/VI (*n* = 812), UMEC (*n* = 804), and salmeterol (*n* = 809) treatment arms (Fig. 1). Overall, 2050/2425 (85%) patients completed the study treatment period. Completion rates were highest for UMEC/VI (717/812 [88%]) compared with UMEC (650/804 [81%]) and salmeterol (683/809 [84%]) with fewer patients in the UMEC/VI group withdrawing consent and withdrawing from the study due to lack of efficacy and other protocol-specified withdrawals (Fig. 1 and Additional file 7: Figure S2). A post hoc assessment demonstrated a decreased risk of premature study withdrawal with UMEC/VI compared with UMEC (HR [95% CI]: 0.60 [0.46, 0.77]; *p* < 0.001) and salmeterol (HR [95% CI]: 0.73 [0.56, 0.96]; *p* = 0.022).

Patient demographics and baseline characteristics were similar between treatment arms (Table 1); mean age was 64.6 years, 988/2425 (41%) were female, and 1203/2424 (50%) were current smokers. Mean post-salbutamol percent predicted FEV_1_ was 55.4% and mean CAT score at baseline was 19.2. Overall, 393/2425 (16%) patients had one moderate exacerbation in the previous year.

**Table 1:** Patient demographics and baseline characteristics

Characteristic	UMEC/VI (*N* = 812)	UMEC (*N* = 804)	SAL (*N* = 809)	Total (*N* = 2425)
Age, years, mean (SD)	64.6 (8.4)	64.9 (8.5)	64.4 (8.5)	64.6 (8.5)
Female, n (%)	319 (39)	327 (41)	342 (42)	988 (41)
Race, n (%)				
White	767 (94)	764 (95)	766 (95)	2297 (95)
Black/African American	24 (3)	23 (3)	25 (3)	72 (3)
American Indian/Alaska Native	13 (2)	12 (1)	12 (1)	37 (2)
Asian	5 (< 1)	1 (< 1)	1 (< 1)	7 (< 1)
Multiple^a^	3 (1)	4 (< 1)	5 (< 1)	12 (< 1)
Current smoker at screening, n (%)	394 (49)	396 (49)	413 (51)	1203 (50)
Smoking pack-years, mean (SD)	49.4 (27.7)	47.6 (25.9)	48.1 (25.8)	48.4 (26.5)
Use of LABD during run-in, n (%)^b^	531 (65)	521 (65)	524 (65)	1576 (65)
LAMA	399 (49)	392 (49)	403 (50)	1194 (49)
LABA	130 (16)	142 (18)	132 (16)	404 (17)
No maintenance medication during run-in, n (%)	250 (31)	250 (31)	249 (31)	749 (31)
Moderate COPD exacerbation history in prior year^c^, n (%)	123 (15)	124 (15)	146 (18)	393 (16)
Duration of COPD, years, mean (SD)	8.8 (6.9)	7.8 (6.0)	8.3 (6.7)	8.3 (6.6)
Post-salbutamol FEV_1_, mL, mean (SD)	1577 (506)	1609 (503)	1600 (523)	1595 (511)
Post-salbutamol % predicted FEV_1_, mean (SD)	54.9 (12.8)	55.9 (12.6)	55.6 (12.8)	55.4 (12.7)
Post-salbutamol FEV_1_/FVC, mean (SD)	0.51 (0.10)	0.52 (0.10)	0.52 (0.10)	0.52 (0.10)
% reversibility to salbutamol, mean (SD)	10.4 (12.8)	10.2 (13.3)	10.7 (13.3)	10.5 (13.1)
GOLD spirometric grade^d^, n (%)				
2	518 (64)	529 (66)	522 (65)	1569 (65)
3	294 (36)	271 (34)	286 (35)	851 (35)
Baseline FEV_1_, mL, mean (SD)	1474 (513)	1503 (505)	1495 (533)	1491 (517)
BDI score, mean (SD)	7.0 (1.8)	7.0 (1.9)	7.1 (1.8)	7.01 (1.9)
Baseline E-RS total score	10.7 (5.6)	10.7 (5.8)	10.4 (5.7)	10.6 (5.7)
Baseline SGRQ score, mean (SD)	44.5 (16.1)	45.0 (16.1)	44.6 (16.3)	44.7 (16.2)
Baseline CAT score, mean (SD)	19.1 (5.9)	19.3 (6.2)	19.3 (6.3)	19.2 (6.1)
Baseline rescue salbutamol, puffs/day, mean (SD)	2.2 (2.6)	2.1 (2.3)	2.2 (2.5)	2.2 (2.5)
Any cardiac comorbidities^e^, n (%)	111 (14)	136 (17)	117 (14)	364 (15)
Any vascular comorbidities^f^, n (%)	444 (55)	434 (54)	448 (55)	1326 (55)

^a^Includes American Indian/Alaska Native and White, Black/African American and White, Native Hawaiian/Other Pacific Islander and White; ^b^patients could be counted for both LAMA and LABA; ^c^number of exacerbations requiring oral or systemic corticosteroids and/or antibiotics (moderate) in 12 months prior to screening (patients with > 1 moderate exacerbation or with a severe exacerbation [requiring hospitalisation] were excluded); ^d^an additional 4 (< 1%) patients with GOLD grade 1 were randomised (UMEC *n* = 3; SAL *n* = 1); ^e^includes coronary artery disease, arrhythmia, congestive heart failure, and myocardial infarction; ^f^includes hypertension and cerebrovascular accident

*BDI* Baseline dyspnoea index, *CAT* COPD Assessment Test, *COPD* Chronic obstructive pulmonary disease, *E-RS* Evaluating Respiratory Symptoms, *FEV*_*1*_ Forced expiratory volume in 1 s, *FVC* Forced vital capacity, *GOLD* Global Initiative for Chronic Obstructive Lung Disease, *LABA* Long-acting β_2_-agonist, *LABD* Long-acting bronchodilator, *LAMA* Long-acting muscarinic antagonists, *SAL* Salmeterol, *SD* Standard deviation, *SGRQ* St George’s Respiratory Questionnaire, *UMEC* Umeclidinium, *VI* Vilanterol

**Fig. 2:**
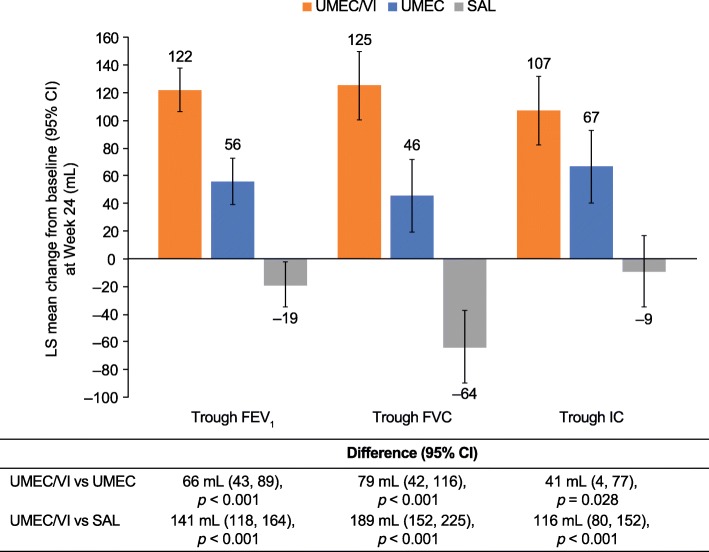
Lung function outcomes. *CI*, confidence interval; *FEV*_*1*_, forced expiratory volume in 1 s; *FVC*, forced vital capacity; *IC*, inspiratory capacity; *LS*, least squares; *SAL*, salmeterol; *UMEC*, umeclidinium; *VI*, vilanterol

The LS mean change from baseline in trough FEV_1_ at Week 24 was 122 mL for UMEC/VI, 56 mL for UMEC, and − 19 mL for salmeterol (Fig. 2). For the primary endpoint, change from baseline in trough FEV_1_ at Week 24 was 66 mL (95% CI: 43, 89) and 141 mL (95% CI: 118, 164) greater with UMEC/VI versus UMEC and salmeterol, respectively (both *p* < 0.001; Fig. 2). The greater improvements in trough FEV_1_ observed with UMEC/VI compared with both monotherapies were consistent across all time points (Additional file 8: Figure S3A). For the other spirometric endpoints (trough FVC and trough IC), UMEC/VI demonstrated significantly greater improvements versus both UMEC and salmeterol at all time points analysed (Fig. 2 and Additional file 8: Figures S3B and S3C). Furthermore, for all spirometric endpoints at Week 24, UMEC demonstrated significantly greater improvements versus salmeterol (Additional file 2: Table S2).

**Fig. 3:**
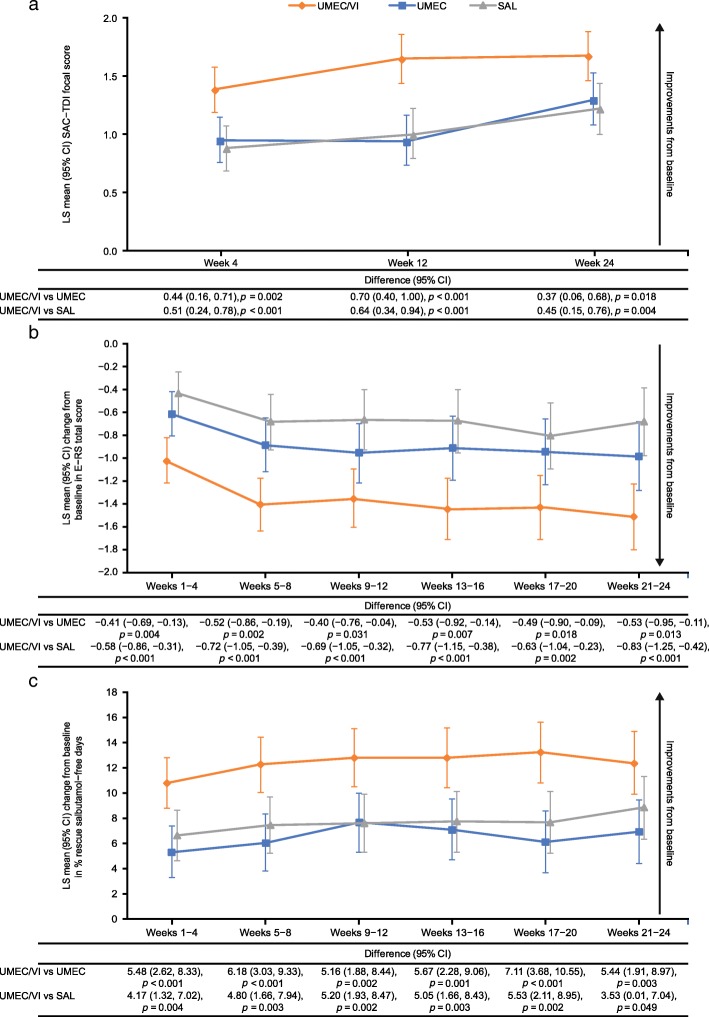
Symptom severity outcomes (SAC-TDI (**a**), E-RS (**b**), % rescue salbutamol-free days (**c**)). *CI*, confidence interval; *COPD*, chronic obstructive pulmonary disease; *E-RS*, Evaluating Respiratory Symptoms-COPD; *LS*, least squares; *SAC-TDI*, self-administered computerised Transition Dyspnoea Index; *SAL*, salmeterol; *UMEC*, umeclidinium; *VI*, vilanterol

Improvements in SAC-TDI score increased over time, with UMEC/VI consistently showing significantly greater improvements versus both monotherapies at all time points (Fig. 3a). All LS mean changes in the three treatment groups exceeded the 1.0 point minimum clinically important difference from baseline in SAC-TDI at Week 24, and responder rates were significantly greater with UMEC/VI versus both UMEC and salmeterol (both *p* < 0.001) (Table 2 and Additional file 3: Table S3). UMEC/VI demonstrated significantly greater improvements in daily respiratory symptoms measured by E-RS total score compared with both monotherapies at all 4-weekly time points analysed (Fig. 3b). The odds of being a responder versus a non-responder in E-RS total score were 1.5 times greater for patients receiving UMEC/VI versus both UMEC and salmeterol (95% CI: 1.2, 1.9 for each treatment) at Weeks 21–24 (both *p* < 0.001) (Table 2). UMEC/VI showed statistically significant improvements at Weeks 21–24 for both LS mean change from baseline and proportion of responders across all E-RS subdomains (breathlessness, cough and sputum, and chest scores) compared with UMEC and SAL, except for LS mean change from baseline in E-RS cough and sputum score versus UMEC, which was not significant (Additional file 4: Table S4). UMEC/VI demonstrated significantly greater improvements from baseline in the percentage of rescue medication-free days and significantly fewer mean inhalations per day compared with either monotherapy over Weeks 1–24 according to the e-diaries (Fig. 3c and Table 2). A greater proportion of patients receiving UMEC/VI (473/707; 67%) rated the overall severity of their COPD as improved from baseline to Week 24 compared with 393/638 (62%) patients receiving UMEC and 413/674 (61%) patients receiving salmeterol. The ordered odds of improvement versus no improvement were significantly higher for patients receiving UMEC/VI compared with UMEC or salmeterol at all time points (all *p* ≤ 0.001) (Table 2 and Additional file 3: Table S3).

**Table 2:** LS mean change from baseline and proportion of responders for patient reported-outcomes

	UMEC/VI (*N* = 812)	UMEC (*N* = 804)	SAL (*N* = 809)
Symptom severity outcomes			
*SAC-TDI focal score at Week 24*			
LS mean (95% CI)	1.68 (1.46, 1.89)	1.30 (1.08, 1.53)	1.22 (1.00, 1.44)
UMEC/VI vs comparator mean difference (95% CI)	–	**0.37 (0.06, 0.68)** ***p*** **= 0.018**	**0.45 (0.15, 0.76)** ***p*** **= 0.004**
SAC-TDI responders^a^, n/N (%)	403/806 (50)	332/799 (42)	330/807 (41)
UMEC/VI vs comparator odds ratio (95% CI)	–	**1.43 (1.17, 1.75);** ***p*** **< 0.001**	**1.48 (1.21, 1.81);** ***p*** **< 0.001**
*E-RS total score at Weeks 21–24*			
LS mean CFB (95% CI)	− 1.52 (− 1.81, − 1.23)	− 0.99 (− 1.29, − 0.69)	− 0.69 (− 0.98, − 0.39)
UMEC/VI vs comparator mean difference (95% CI)	–	**− 0.53 (− 0.95, − 0.11)** ***p*** **= 0.013**	**− 0.83 (− 1.25, − 0.42)** ***p*** **< 0.001**
E-RS responders^b^, n/N (%)	290/809 (36)	219/800 (27)	217/808 (27)
UMEC/VI vs comparator odds ratio (95% CI)	–	**1.52 (1.22, 1.89);** ***p*** **< 0.001**	**1.53 (1.23, 1.90);** ***p*** **< 0.001**
*Rescue medication use at Weeks 1–24*			
Mean inhalations/day			
LS mean CFB (95% CI)	− 0.61 (− 0.71, − 0.50)	− 0.28 (− 0.38, − 0.17)	− 0.32 (− 0.43, − 0.22)
UMEC/VI vs comparator mean difference (95% CI)	–	**− 0.33 (− 0.48, − 0.18)** ***p*** **< 0.001**	**− 0.28 (− 0.43, − 0.14)** ***p*** **< 0.001**
% rescue medication-free days			
LS mean CFB (95% CI)	12.39 (10.28, 14.50)	6.55 (4.42, 8.68)	7.68 (5.55, 9.80)
UMEC/VI vs comparator mean difference (95% CI)	–	**5.84 (2.84, 8.84)** ***p*** **< 0.001**	**4.71 (1.72, 7.70)** ***p*** **= 0.002**
*Global assessment of disease severity*^c^ *at Week 24*			
UMEC/VI vs comparator ordered odds ratio for improvement in response category (95% CI)	–	**1.38 (1.14, 1.67);** ***p*** **= 0.001**	**1.38 (1.14, 1.68);** ***p*** **< 0.001**
Health status outcomes			
*SGRQ total score at Week 24*			
LS mean CFB (95% CI)	− 4.98 (− 5.89, − 4.07)	− 5.23 (− 6.18, − 4.28)	− 3.29 (− 4.22, − 2.36)
UMEC/VI vs comparator mean difference (95% CI)	–	0.25 (− 1.07, 1.57) *p* = 0.709	**− 1.69 (− 2.99, − 0.39)** ***p*** **= 0.011**
SGRQ responders^d^, n/N (%)	366/811 (45)	329/802 (41)	291/809 (36)
UMEC/VI vs comparator odds ratio (95% CI)	–	1.21 (0.99, 1.48); *p* = 0.063	**1.49 (1.22, 1.83);** ***p*** **< 0.001**
*CAT score at Week 24*			
LS mean CFB (95% CI)	− 3.5 (− 3.9, − 3.1)	− 3.4 (− 3.9, − 3.0)	− 2.9 (− 3.4, − 2.5)
UMEC/VI vs comparator mean difference (95% CI)	–	0.0 (− 0.6, 0.6) *p* = 0.891	− 0.5 (− 1.1, 0.1) *p* = 0.074
CAT responders^e^, n/N (%)	447/812 (55)	385/804 (48)	406/809 (50)
UMEC/VI vs comparator odds ratio (95% CI)	–	**1.35 (1.11, 1.65);** ***p*** **= 0.003**	**1.23 (1.01, 1.50);** ***p*** **= 0.037**

^a^SAC-TDI responders were defined as a ≥ 1-unit improvement from baseline; ^b^E-RS responders were defined as a reduction of ≥2 from baseline; ^c^overall assessment of change in COPD severity was rated using a seven-point Likert scale (‘Much Better’, ‘Slightly Better’, ‘Better’, ‘No Change’, ‘Slightly Worse’, ‘Worse’, ‘Much Worse’). Ordered response ratios were reported as odds of better response category; ^d^SGRQ responders were defined as a ≥ 4-point reduction from baseline; ^e^CAT responders were defined as a ≥ 2-unit improvement from baseline

*CAT* COPD Assessment Test, *CFB* Change from baseline, CI, *COPD* Chronic obstructive pulmonary disease; e-diary, electronic diary, *E-RS* Evaluating Respiratory Symptoms-COPD, *LS* Least squares, *n/N* number of responders/number of patients with analysable data, *SAC-TDI* Self-administered computerised Transition Dyspnoea Index, *SAL* Salmeterol, *SGRQ* St George’s Respiratory Questionnaire, *UMEC* Umeclidinium, *VI* Vilanterol

When considering symptomatic PROs, there were no differences between UMEC and salmeterol for SAC-TDI, E-RS total score, rescue medication use, or the ordered odds of improvement in patients’ global assessment of disease severity at the final assessment period (Additional file 2: Table S2) or at any other time point.

Statistically significant improvements in the odds of being a responder versus a non-responder in SGRQ were observed with UMEC/VI versus salmeterol at all time points (OR: 1.27–1.49, *p* < 0.05), and at Weeks 4 and 12 versus UMEC (OR: 1.33–1.34, *p* < 0.01) (Table 2 and Additional file 3: Table S3). UMEC/VI demonstrated clinically meaningful improvements in LS mean change from baseline in SGRQ total score over Weeks 12–24. Statistically significant improvements with UMEC/VI versus salmeterol were observed at all time points (all *p* < 0.05); however, statistically significant improvements were not observed versus UMEC (Table 2 and Additional file 9: Figure S4A). UMEC/VI demonstrated significantly greater improvements in the proportion of CAT responders versus UMEC at Week 12 (*p* < 0.05) and versus both UMEC and salmeterol at Week 24 (both *p* < 0.05) (Table 2 and Additional file 3: Table S3). Similar improvements in LS mean change from baseline CAT total scores over 24 weeks were seen for all treatment groups, with no significant differences from baseline with UMEC/VI versus either monotherapy at any time point, except for versus salmeterol at Week 12 (Additional file 9: Figure S4B). When considering UMEC versus salmeterol at Week 24, statistical significance was achieved for SGRQ, which favoured UMEC in terms of both proportion of responders (*p* = 0.045) and LS mean difference (*p* = 0.004); however, no significant difference was observed at other time points, or on CAT score or response rate at any time point (Additional file 2: Table S2).

**Fig. 4:**
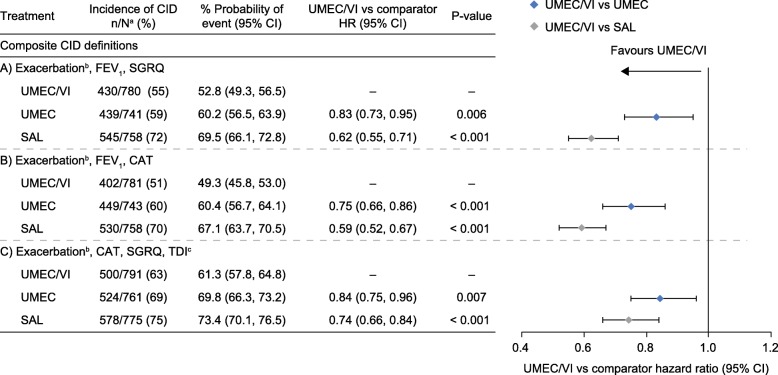
Risk of a first CID up to Day 168 across multiple composite definitions. **a** N, patients with at least 1 post baseline assessment (not including exacerbations) for at least one of the individual components or patients who had an exacerbation; **b** moderate/severe exacerbation; **c** assessed using a self-administered computerised version. *CAT*, COPD Assessment Test; *CI*, confidence interval; *CID*, clinically important deterioration; *COPD*, chronic obstructive pulmonary disease; *FEV*_*1*_ trough forced expiratory volume in 1 s; *HR*, hazard ratio; *n*, number of patients with an event; *TDI*, Transition Dyspnoea Index; *SAL*, salmeterol; *SGRQ*, St George’s Respiratory Questionnaire; *UMEC*, umeclidinium; *VI*, vilanterol

**Fig. 5:**
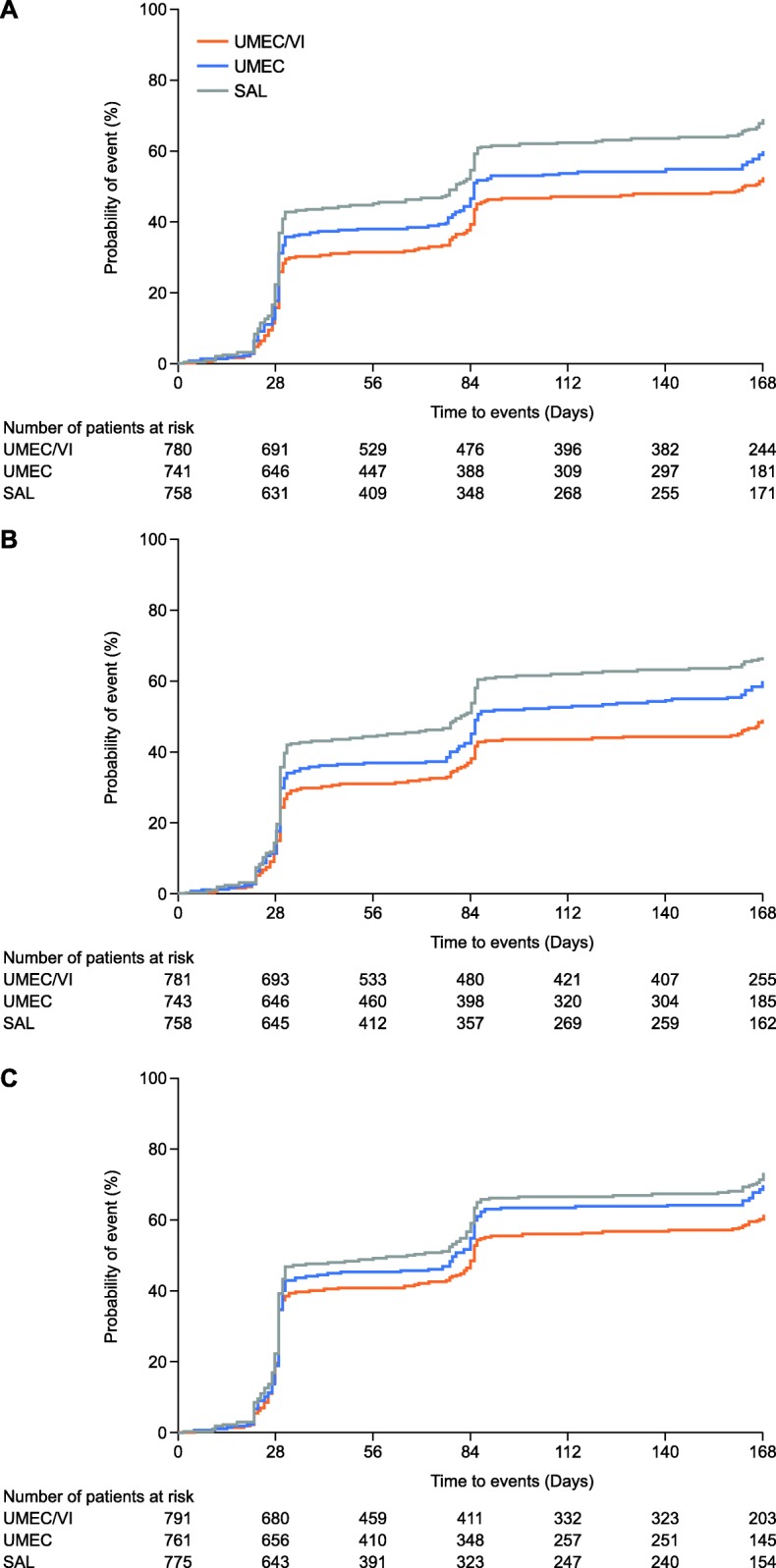
Kaplan–Meier plots of time to first CID for three definitions^a^. ^a^CID was defined as: **a** – a first moderate or severe exacerbation, and/or a trough FEV_1_ decrease from baseline of ≥100 mL, and/or a deterioration in SGRQ ≥4 units from baseline; **b** – a first moderate or severe exacerbation, and/or a trough FEV_1_ decrease from baseline of ≥100 mL, and/or a deterioration in CAT ≥2 units from baseline; **c** – a first moderate or severe exacerbation, and/or a deterioration in SGRQ ≥4 units from baseline and/or a deterioration in CAT ≥2 units from baseline and/or a TDI deterioration ≥1 unit decrease from baseline. *CAT*, COPD Assessment Test; *CID*, clinically important deterioration; *FEV*_*1*_, forced expiratory volume in 1 s; *SAL*, salmeterol; *SGRQ*, St George’s Respiratory Questionnaire; *TDI*, transition dyspnoea index; *UMEC*, umeclidinium; *VI*, vilanterol

Overall, the probability of an individual patient experiencing a CID within 24 weeks ranged between 49 and 73% for all treatments across the different composite definitions, with a consistently lower probability in patients receiving UMEC/VI compared with UMEC and salmeterol (Fig. 4). For all CID definitions, including the FEV_1_-free definition, fewer CID events were observed with UMEC/VI, demonstrating increased protection from COPD deteriorations compared with both monotherapies (Fig. 5). The probability of having a moderate/severe exacerbation CID event up to Day 168 was low across all treatments (Additional file 10: Figure S5 and Additional file 11: Figure S6). Overall, 363 patients experienced a moderate (304/363 [83.7%]) or severe (59/363 [16.3%]) exacerbation (UMEC/VI: 101/812 [12%]; UMEC: 116/804 [14%]; salmeterol: 146/809 [18%]). The probability (95% CI) of a first moderate or severe exacerbation to Day 168 was 12.8% (10.7, 15.4), 16.1% (13.6, 19.0), and 19.4% (16.7, 22.4) for UMEC/VI, UMEC, and salmeterol, respectively. The HR (95% CI) for risk of a first moderate or severe exacerbation was 0.81 (0.62, 1.05; *p* = 0.114) with UMEC/VI versus UMEC, and 0.64 (0.50, 0.83; *p* < 0.001) with UMEC/VI versus salmeterol. There was no significant difference in the occurrence of severe exacerbations between treatment groups. Patients were less likely to experience a moderate or severe exacerbation compared with the probability of having one of the other CID component events (trough FEV_1_, SGRQ, CAT, TDI; UMEC/VI [22–36%], UMEC [31–39%], and salmeterol 36–43%]) (Additional file 10: Figure S5 and Additional file 11: Figure S6). UMEC/VI significantly reduced the risk of all individual CID component events versus salmeterol (*p* < 0.05), and the FEV_1_ and TDI components versus UMEC (*p* < 0.001) (Additional file 10: Figure S5 and Additional file 11: Figure S6).

The probability of experiencing a short-term CID within 24 weeks was lower for UMEC compared with salmeterol for all three CID definitions (Additional file 2: Table S2). For individual component CID events, UMEC significantly reduced the risk of FEV_1_ events versus salmeterol (*p* < 0.001); however, significant differences were not observed for other individual CID component events (Additional file 2: Table S2).

The overall incidence of on-treatment AEs and SAEs was similar across treatment groups, and the incidence of drug-related AEs was low (≤5%) (Table 3). The types of AEs observed were representative of known effects of anticholinergics or β_2_-agonists, with the most frequent AE in all treatment groups being nasopharyngitis (≤11%). Incidence of non-fatal SAEs was similar across treatment groups (4–6%) and none were considered drug-related. A total of eight fatal SAEs were reported, four each in the UMEC/VI (any cardiac disorder: *n* = 3, and pneumonia: *n* = 1) and UMEC (any cardiac disorder: n = 1, and any respiratory, thoracic and mediastinal disorder: n = 3) arms; none were considered drug-related by the investigators. Consistent with previous studies, the incidence of fatal cardiovascular SAEs was < 1% in all treatment groups.

**Table 3:** Adverse events

	UMEC/VI (*N* = 812)	UMEC (*N* = 804)	SAL (*N* = 809)
AE, n (%)			
AE	315 (39)	316 (39)	314 (39)
Drug-related AE	29 (4)	37 (5)	27 (3)
AE leading to study withdrawal	32 (4)	36 (4)	26 (3)
SAE, n (%)			
Non-fatal SAE	46 (6)	31 (4)	38 (5)
Drug-related non-fatal SAE	0	0	0
Fatal SAE^a^	4 (< 1)	4 (< 1)	0
Drug-related fatal SAE	0	0	0
Most frequent AEs^a^, n (%)			
Nasopharyngitis	68 (8)	87 (11)	84 (10)
Upper respiratory tract infection	19 (2)	12 (1)	20 (2)
Influenza	20 (2)	9 (1)	18 (2)
Back pain	10 (1)	13 (2)	15 (2)
Cough	14 (2)	11 (1)	10 (1)
Headache	10 (1)	17 (2)	6 (< 1)

^a^The incidence of fatal cardiovascular SAEs was < 1% in all treatment groups, with three cardiac disorders observed in the UMEC/VI arm and one in the UMEC arm (one acute myocardial infarction in each treatment group). ^b^includes all on-treatment AEs occurring in ≥2% of any treatment group

*AE* Adverse event, *SAE* Serious adverse event, *SAL* Salmeterol, *UMEC* Umeclidinium, *VI* Vilanterol

## Discussion

The EMAX trial is the largest 24-week RCT to date to compare dual- versus mono-bronchodilator therapies in symptomatic patients with COPD who were not receiving ICS either at baseline or concurrently during the study treatment period. It is also the first prospective assessment of the composite CID endpoint as a marker of short-term disease worsening and treatment failure, in patients receiving LAMA/LABA combination therapy compared with LAMA and LABA monotherapies. The study provides consistent evidence that confirms the known incremental benefits of dual bronchodilation on lung function compared with mono-bronchodilator therapy [2, 5, 6, 8] and extends current knowledge by providing a detailed assessment of the symptomatic benefits of dual bronchodilation and its potential to reduce treatment failure in symptomatic COPD.

UMEC/VI demonstrated consistent early, sustained, and similar improvements for all symptomatic outcomes compared with UMEC and salmeterol. These symptom improvements appear not to fully relate to the level of spirometry improvements versus the monotherapies, where a ≥ 2-fold higher magnitude of spirometry improvement was observed for UMEC/VI versus salmeterol compared with UMEC/VI versus UMEC. In contrast, the level of symptom benefits observed when comparing UMEC/VI versus UMEC and versus salmeterol were broadly comparable. In addition, despite consistent improvements in all spirometry parameters in favour of UMEC versus salmeterol, no symptom benefits were observed between the monotherapies at any time point. These findings indicate that the differences in trough spirometry observed between the treatment arms may not capture the inherent daily variability of airway tone and fluctuations in symptoms recalled by patients over longer periods of assessment. Nevertheless, these findings support the current GOLD strategy document, which indicates no overall preference for LAMA or LABA therapy in symptomatic low-risk patients [1].

This study is the first to prospectively assess the CID composite endpoint as a measure of disease stability and freedom from short-term disease worsening/treatment failure in symptomatic patients receiving three different long-acting bronchodilator therapies. Previous studies have retrospectively tested different CID definitions to better understand the heterogeneity of short-term worsening/treatment failure [8, 13–16], whereas this study has prospectively assessed three different definitions including one focused exclusively on PROs that does not include a decrease in FEV_1_. Previous studies have shown that more than half of patients receiving mono-bronchodilator therapy experience short-term worsening as measured by CID over 24 weeks, a finding confirmed in this trial. In keeping with previous post hoc analyses, this prospective study also shows that the risk of a first CID is reduced by dual-bronchodilator maintenance therapy [8, 13–15, 22]. In this study, UMEC/VI consistently provided increased protection from early treatment failure versus UMEC and salmeterol across all three composite CID definitions examined, including the CID definition that excluded a trough FEV_1_ decrease. Together, these findings provide additional evidence to support early intensification of bronchodilation in symptomatic patients with COPD, before a high exacerbation risk develops. In addition, this study provides further evidence that the concept of the composite CID endpoint has the potential to monitor individual patients following initiation of standard of care therapy to determine future prognosis. Indeed, short-term CID and lack of disease stability, using FEV_1_, SGRQ and exacerbations, have been demonstrated to be predictors of sustained long-term deterioration and poor clinical outcomes, including an increase in hospital admissions and mortality over 3–4 years [13, 22–24].

UMEC/VI achieved significant reductions in the risk of moderate or severe exacerbations versus salmeterol, but not versus UMEC. These findings are compatible with a meta-analysis of previous bronchodilator studies, which demonstrated clear benefits in reducing exacerbation risk with dual therapy versus LABAs, yet inconclusive findings for dual therapies versus LAMAs [5]. Likewise, our findings are consistent with results from other studies that evaluated the effect of dual-bronchodilator therapy in reducing exacerbation risk versus LAMA monotherapy in populations at increased exacerbation risk [9, 10]. In contrast to the current study, these trials all allowed continued use of ICS alongside LAMA/LABA or LAMA therapy. Therefore, the absence of a conclusive add-on LABA effect to LAMA bronchodilation in patients at low or high risk of exacerbations cannot be confirmed, either in the absence or presence of ICS. In the absence of large, long-term exacerbation studies of initation of long-acting bronchodilator therapy in patients not receiving ICS, there remains a need to quantify what may be a small benefit in exacerbation protection in these patients and to determine whether it is clinically useful.

All treatments were well-tolerated with no unexpected AEs, a finding in line with previous studies [7, 25, 26]. The greater efficacy of UMEC/VI compared with UMEC and salmeterol was seen with no increase in safety concerns compared with monotherapies. Additionally, more patients on UMEC/VI were able to complete the 24-week study, providing further indication of treatment benefit, including tolerability and protection from deterioration, with UMEC/VI compared with LAMA and LABA monotherapies. It should be noted that fatal SAEs were only observed in treatment arms containing UMEC; however, incidence was < 1% and consistent with the incidence observed in previous studies [7] and no SAEs were considered to be drug-related by the investigators.

A major strength of this study is that it is the first and largest RCT comparing dual bronchodilation versus monotherapy in symptomatic patients with COPD who were not receiving ICS and it thus fills an important knowledge gap. Patients who are not receiving concurrent ICS therapy may be considered to have early, less severe COPD and may be ideal candidates for early optimisation to dual bronchodilation. Our comprehensive assessment of the clinical impact of dual bronchodilation with a LAMA/LABA indicates consistent additional benefits over monotherapy across a range of clinical and functional outcomes including preventing meaningful treatment failure. Unlike studies in high-risk patients, the role of optimising care in symptomatic patients with low risk of exacerbations has been neglected. Consequently, the timing of intensification of care in such patients to prevent future poor outcomes remains uncertain and many patients on long-acting bronchodilator monotherapy continue to experience significant symptoms [27]. The high baseline CAT scores observed in this analysis are in keeping with those reported in patients who experience frequent exacerbations [9, 28]. Consequently, it is important for physicians to recognise that patients who may not experience frequent exacerbations still face a significant impact on their health and wellbeing, and that the disease burden in low exacerbation risk patients should not be underestimated. Indeed, the symptomatic burden at baseline in this population, who would be considered as typical GOLD group B patients, has been shown to be associated with a higher future risk of hospitalised exacerbations and mortality compared with GOLD group A patients [29, 30]. Our findings suggest that there is a need to consider initiation of bronchodilation with LAMA/LABAs, or early escalation to dual therapy, in this symptomatic patient population.

There are potential limitations to consider in interpreting this study. It was powered for the primary endpoint (trough FEV_1_) and for the secondary endpoint SAC-TDI at Week 24 but was not powered to detect differences in other PROs. The failure to include both monocomponents of UMEC/VI as comparator arms may be perceived as a limitation of this study; however, this was necessitated by local country requirements to generate new data only against currently available LAMA and LABA therapies. Unfortunately, VI is not available as a licensed drug; therefore, SAL was instead selected as a comparator LABA for this study. We note that although SAL provided similar efficacy to UMEC on the majority of PROs, a deterioration in FEV_1_ was observed at Week 24 in patients receiving SAL. This suggests that SAL may have been less effective at sustaining 24 h bronchodilation overtime than the baseline LABA or LAMA agents that patients were using at study entry. Furthermore, at the time the EMAX study was designed there were insufficient data available to power the study on each CID component type. Additionally, whilst several composite measures of CID were used in this analysis, including a definition that excluded deterioration in lung function, further research is needed to reach a consensus on how to achieve disease stability in COPD and how best to monitor short-term deterioration/treatment failure in individual patients.

## Conclusions

In symptomatic, low exacerbation risk patients with COPD who were not receiving ICS, once-daily UMEC/VI provided consistent early and sustained improvements in lung function and symptoms and reduced the probability of short-term COPD worsening compared with both UMEC and salmeterol monotherapies, with no additional safety concerns. Our findings suggest that a sizeable group of symptomatic patients may gain important benefits from earlier use of dual versus mono bronchodilators. Research is now required to help predict the patient type most well-suited for early use of dual therapy.

## Supplementary information

**Additional file 1:** **Table S1.** Patient-reported outcomes. ^a^SAC-TDI responders were defined as a ≥ 1-unit improvement from baseline; ^b^total score and subscales (breathlessness, cough and sputum, chest); ^c^E-RS responders were defined as a reduction of ≥2 from baseline; ^d^SGRQ responders were defined as a ≥ 4-point reduction from baseline; ^e^CAT responders were defined as a  ≥ 2-unit improvement from baseline. CAT, COPD Assessment Test; COPD, chronic obstructive pulmonary disease; E-RS, Evaluating Respiratory Symptoms-COPD; LS, least squares; SAC-TDI, self-administered computerised-Transition Dyspnoea Index; SGRQ, St George’s Respiratory Questionnaire.

**Additional file 2:** **Table S2.** UMEC versus salmeterol comparisons for all outcomes. ^a^Symptom severity outcomes presented for Week 24 for SAC-TDI and global assessment of disease severity, for Weeks 21–24 for E-RS, and for Weeks 1–24 for rescue salbutamol use; ^b^data are LS mean (95% CI); ^c^SAC-TDI responders were defined as a ≥ 1-unit improvement from baseline; ^d^E-RS responders were defined as a reduction of ≥2 from baseline; ^e^overall assessment of change in COPD severity was rated using a seven-point Likert scale (‘Much Better’, ‘Slightly Better’, ‘Better’, ‘No Change’, ‘Slightly Worse’, ‘Worse’, ‘Much Worse’). Ordered response ratios were reported as odds of better response category; ^f^SGRQ responders were defined as a ≥ 4-point reduction from baseline; ^g^CAT responders were defined as a ≥ 2-unit improvement from baseline. CAT, COPD Assessment Test; CI, confidence interval; CID, clinically important deterioration; COPD, chronic obstructive pulmonary disease; E-RS, Evaluating Respiratory Symptoms-COPD; FEV_1_, trough forced expiratory volume in 1 sec; LS, least squares; n, number of responders/patients with an event; N, number of patients with analysable data; SAC-TDI, self-administered computerised Transition Dyspnoea Index; SAL, salmeterol; SGRQ, St George’s Respiratory Questionnaire; UMEC, umeclidinium; VI, vilanterol.

**Additional file 3:** **Table S3.** Proportion of responders for symptom severity and health status outcomes – additional timepoints. ^a^SAC-TDI responders were defined as a ≥ 1-unit improvement from baseline; ^b^E-RS responders were defined as a reduction of ≥2 from baseline; ^c^overall assessment of change in COPD severity was rated using a seven-point Likert scale (‘Much Better’, ‘Slightly Better’, ‘Better’, ‘No Change’, ‘Slightly Worse’, ‘Worse’, ‘Much Worse’). Ordered response ratios were reported as odds of better response category; ^d^SGRQ responders were defined as a ≥ 4-point reduction from baseline; ^e^CAT responders were defined as a ≥ 2-unit improvement from baseline. CAT, COPD Assessment Test; CI, confidence interval; COPD, chronic obstructive pulmonary disease; e-diary, electronic diary; E-RS, Evaluating Respiratory Symptoms-COPD; n/N, number of responders/number of patients with analysable data; SAC-TDI, self-administered computerised Transition Dyspnoea Index; SAL, salmeterol; SGRQ, St George’s Respiratory Questionnaire; UMEC, umeclidinium; VI, vilanterol.

**Additional file 4:** **Table S4.** LS mean change from baseline and proportion of responders^a^ for E-RS subdomains at Weeks 21–24. ^a^E-RS responders were defined as a reduction of ≥1 unit from baseline for E-RS breathlessness score, and a reduction of ≥0.7 units from baseline for cough and sputum, and chest scores. CI, confidence interval; CFB, change from baseline; E-RS, Evaluating Respiratory Symptoms-COPD; LS, least squares; n/N, number of responders/number of patients with analysable data; SAL, salmeterol; UMEC, umeclidinium; VI, vilanterol.

**Additional file 5:** **Table S5.** List of investigators.

**Additional file 6:** **Figure S1.** Study design. ^a^Pre-screening, existing bronchodilator maintenance therapy was limited to a LAMA or LABA, with patients required to be ICS and ICS/LABA free for ≥6 weeks and LAMA/LABA free for ≥2 weeks prior to run-in. ^b^Patients were permitted to continue use of inhaled LAMAs or LABAs and/or study-provided as-needed salbutamol during the run-in period. BID, twice daily; DPI, dry powder inhaler; LABA, long-acting β_2_-agonist; LAMA, long-acting muscarinic antagonist; QD, once daily; R, randomisation; SAL, salmeterol; UMEC, umeclidinium; V, visit; VI, vilanterol.

**Additional file 7:** **Figure S2.** Kaplan–Meier curve time to withdrawal. Note: post hoc analyses showed that 12, 19, and 16% of patients receiving UMEC/VI, UMEC, and SAL withdrew from study treatment; UMEC/VI versus UMEC HR (95% CI): 0.60 (0.46, 0.77), *p* < 0.001; UMEC/VI versus SAL HR (95% CI): 0.73 (0.56, 0.96), *p* = 0.022. SAL, salmeterol; UMEC, umeclidinium; VI, vilanterol.

**Additional file 8:** **Figure S3.** Lung function outcomes (trough FEV_1_ [A], FVC [B], IC [C]). CI, confidence interval; FEV_1_, forced expiratory volume in 1 s; FVC, forced vital capacity; IC, inspiratory capacity; LS, least squares; SAL, salmeterol; UMEC, umeclidinium; VI, vilanterol.

**Additional file 9:** **Figure S4.** Health status outcomes (SGRQ score [A], CAT score [B]). CAT, COPD assessment test; CI, confidence interval; COPD, chronic obstructive pulmonary disease; LS least squares; SGRQ, St George’s Respiratory Questionnaire; SAL, salmeterol; UMEC, umeclidinium; VI, vilanterol.

**Additional file 10:** **Figure S5.** Probability of patients experiencing individual CID components to Day 168. CAT, COPD Assessment Test; CI, confidence interval; CID, clinically important deterioration; FEV_1_ trough forced expiratory volume in 1 s; HR, hazard ratio; n/N, number of patients with an event/number of patients with analysable data; TDI, Transition Dyspnoea Index; SAL, salmeterol; SGRQ, St George’s Respiratory Questionnaire; UMEC, umeclidinium; VI, vilanterol.

**Additional file 11:** **Figure S6.** Kaplan–Meier plots of time to first CID event^a^. ^a^Panel A: first on-treatment moderate/severe exacerbation; Panel B: first decrease from baseline of ≥100 mL in trough FEV_1_; Panel C: first increase from baseline of ≥4 units in SGRQ total score; Panel D: first increase from baseline of ≥2 units in CAT score; Panel E: first decrease of ≥1 unit in SAC-TDI. CAT, COPD Assessment Test; CID, clinically important deterioration; FEV_1_, forced expiratory volume in 1 s; SAL, salmeterol; SGRQ, St George’s Respiratory Questionnaire; TDI, transition dyspnoea index; UMEC, umeclidinium; VI, vilanterol.

## Data Availability

Anonymised individual participant data and study documents can be requested for further research from www.clinicalstudydatarequest.com.

## Abbreviations

AEs: Adverse events
BDI: Baseline dyspnoea index
CAT: COPD Assessment Test
CID: Clinically important deterioration
CIs: Confidence intervals
COPD: Chronic Obstructive Pulmonary Disease
E-diary: Electronic diary
EMAX: Early MAXimisation of bronchodilation for improving COPD stability
E-RS: Evaluating Respiratory Symptoms
FEV_1_: Forced expiratory volume in 1 s
FVC: Forced vital capacity
GOLD: Global Initiative for Chronic Obstructive Lung Disease
HR: Hazard ratios
IC: Inspiratory capacity
ICS: Inhaled corticosteroids
ITT: Intent-to-treat
LABAs: Long-acting β_2_-agonists
LAMAs: Long-acting muscarinic antagonists
LS: Least squares
OR: Odds ratios
PROs: Patient-reported outcomes
RCTs: Randomised controlled trials
SAC-TDI: Self-administered computerised-Transition Dyspnoea Index
SAEs: Serious adverse events
SAL: Salmeterol
SD: Standard deviation
SGRQ: St George’s Respiratory Questionnaire
UMEC: Umeclidinium
UMEC/VI: Umeclidinium/vilanterol
