# Supplementary material for: Efficacy of umeclidinium/vilanterol versus umeclidinium and salmeterol monotherapies in symptomatic patients with COPD not receiving inhaled corticosteroids: the EMAX randomised trial
Source: Respir Res. 2019 Oct 30;20:238. doi: 10.1186/s12931-019-1193-9 (PMC6821007; doi:10.1186/s12931-019-1193-9)
Supplement: Supplementary file 1 — Additional file 1: Table S1. Patient-reported outcomes. aSAC-TDI responders were defined as a ≥ 1-unit improvement from baseline; btotal score and subscales (breathlessness, cough and sputum, chest); cE-RS responders were defined as a reduction of ≥2 from baseline; dSGRQ responders were defined as a ≥ 4-point reduction from baseline; eCAT responders were defined as a ≥ 2-unit improvement from baseline. CAT, COPD Assessment Test; COPD, chronic obstructive pulmonary disease; E-RS, Evaluating Respiratory Symptoms-COPD; LS, least squares; SAC-TDI, self-administered computerised-Transition Dyspnoea Index; SGRQ, St George’s Respiratory Questionnaire. [file 12931_2019_1193_MOESM1_ESM.docx]

**Additional Table 1** Patient-reported outcomes

| **Patient-reported outcome** | **Measurement frequency** |
| --- | --- |
| **Symptom severity outcomes** |  |
| *SAC-TDI* |  |
| LS mean SAC-TDI | At study visits (Weeks 4, 12, and 24) |
| SAC-TDI responders^a^ |  |
| *E-RS*^b^ |  |
| LS mean change from baseline in E-RS | Daily (reported as 4-weekly means and over Weeks 1–24) |
| E-RS responders^c^ |  |
| *Rescue salbutamol use* |  |
| Percentage of rescue salbutamol-free days | Daily (reported as 4-weekly means and over Weeks 1–24) |
| Mean number of inhalations/day |  |
| *Global assessment of disease severity* |  |
| LS mean change from baseline in global assessment of disease severity | At study visits (Weeks 4, 12, and 24) |
| **Health status outcomes** |  |
| *SGRQ score* |  |
| LS mean change from baseline in SGRQ | At study visits (Weeks 4, 12, and 24) |
| SGRQ responders^d^ |  |
| *CAT score* |  |
| LS mean change from baseline in CAT score | At study visits (Weeks 4, 12, and 24) |
| CAT responders^e^ |  |

^a^SAC-TDI responders were defined as a ≥1-unit improvement from baseline; ^b^total score and subscales (breathlessness, cough and sputum, chest); ^c^E-RS responders were defined as a reduction of ≥2 from baseline; ^d^SGRQ responders were defined as a ≥4-point reduction from baseline; ^e^CAT responders were defined as a
≥2-unit improvement from baseline.

CAT, COPD Assessment Test; COPD, chronic obstructive pulmonary disease; E-RS, Evaluating Respiratory Symptoms-COPD; LS, least squares; SAC-TDI, self-administered computerised-Transition Dyspnoea Index; SGRQ, St George’s Respiratory
