# Supplementary material for: Efficacy of umeclidinium/vilanterol versus umeclidinium and salmeterol monotherapies in symptomatic patients with COPD not receiving inhaled corticosteroids: the EMAX randomised trial
Source: Respir Res. 2019 Oct 30;20:238. doi: 10.1186/s12931-019-1193-9 (PMC6821007; doi:10.1186/s12931-019-1193-9)
Supplement: Supplementary file 2 — Additional file 2: Table S2. UMEC versus salmeterol comparisons for all outcomes. aSymptom severity outcomes presented for Week 24 for SAC-TDI and global assessment of disease severity, for Weeks 21–24 for E-RS, and for Weeks 1–24 for rescue salbutamol use; bdata are LS mean (95% CI); cSAC-TDI responders were defined as a ≥ 1-unit improvement from baseline; dE-RS responders were defined as a reduction of ≥2 from baseline; eoverall assessment of change in COPD severity was rated using a seven-point Likert scale (‘Much Better’, ‘Slightly Better’, ‘Better’, ‘No Change’, ‘Slightly Worse’, ‘Worse’, ‘Much Worse’). Ordered response ratios were reported as odds of better response category; fSGRQ responders were defined as a ≥ 4-point reduction from baseline; gCAT responders were defined as a ≥ 2-unit improvement from baseline. CAT, COPD Assessment Test; CI, confidence interval; CID, clinically important deterioration; COPD, chronic obstructive pulmonary disease; E-RS, Evaluating Respiratory Symptoms-COPD; FEV1, trough forced expiratory volume in 1 sec; LS, least squares; n, number of responders/patients with an event; N, number of patients with analysable data; SAC-TDI, self-administered computerised Transition Dyspnoea Index; SAL, salmeterol; SGRQ, St George’s Respiratory Questionnaire; UMEC, umeclidinium; VI, vilanterol. [file 12931_2019_1193_MOESM2_ESM.docx]

**Additional Table 2** UMEC versus salmeterol comparisons for all outcomes

|  | **UMEC  (N=804)** | **SAL  (N=809)** | **Mean difference**  **(95% CI); p-value** |
| --- | --- | --- | --- |
| **LS mean change from baseline (95% CI) analyses** | | | |
| *Lung function outcomes at Week 24* | | | |
| Trough FEV_1_ | 56 (39, 73) | –19 (–35, –2) | 75 (51, 98); p<0.001 |
| Trough FVC | 46 (19, 72) | –64 (–90, –37) | 109 (72, 147); p<0.001 |
| Trough IC | 67 (40, 93) | –9 (–35, 17) | 76 (39, 112); p<0.001 |
| *Symptom severity outcomes^a^* | | | |
| SAC-TDI focal score^b^ | 1.30 (1.08, 1.53) | 1.22 (1.00, 1.44) | 0.08 (–0.23, 0.39); p=0.61 |
| E-RS total score | –0.99 (–1.29, –0.69) | –0.69 (–0.98, –0.39) | –0.30 (–0.72, 0.12); p=0.16 |
| Rescue salbutamol use, % free days | 6.55 (4.42, 8.68) | 7.68 (5.55, 9.80) | –1.13 (–4.14, 1.88); p=0.46 |
| Rescue salbutamol use, mean, puffs/day | –0.28 (–0.38, –0.17) | –0.32 (–0.43, –0.22) | 0.05 (–0.10, 0.19); p=0.52 |
| *HRQoL outcomes at Week 24* | | | |
| SGRQ | –5.23 (–6.18, –4.28) | –3.29 (–4.22, –2.36) | –1.94 (–3.27, –0.61); p=0.004 |
| CAT | –3.4 (–3.9, –3.0) | –2.9 (–3.4, –2.5) | –0.5 (–1.1, 0.1); p=0.11 |
|  | **UMEC  (N=804)** | **SAL  (N=809)** | **Odds ratio**  **(95% CI); p-value** |
| **Responder analyses** | | | |
| *Symptom severity outcomes* |  |  |  |
| SAC-TDI responders,^c^ n/N (%) | 332/799 (42) | 330/807 (41) | 1.03 (0.84, 1.27); p=0.76 |
| E-RS total score responders,^d^ n/N (%) | 219/800 (27) | 217/808 (27) | 1.00 (0.80, 1.26); p=0.97 |
| Global assessment of disease severity^e^ | – | – | 1.00 (0.82, 1.22); p=0.97 |
| *Health status outcomes at Week 24* | | | |
| SGRQ responders,^f^ n/N (%) | 329/802 (41) | 291/809 (36) | 1.23 (1.00, 1.51); p=0.045 |
| CAT responders,^g^ n/N (%) | 385/804 (48) | 406/809 (50) | 0.91 (0.75, 1.11); p=0.36 |
|  | **UMEC  (N=804)** | **SAL  (N=809)** | **Hazard ratio**  **(95% CI); p-value** |
| **Time to first exacerbation** | | | |
| Moderate or severe exacerbation, % probability of an event to Day 168 (95% CI) | 16.1 (13.6, 19.0) | 19.4 (16.7, 22.4) | 0.80 (0.62, 1.02); p=0.067 |
| Severe exacerbation, % probability of an event to Day 168 (95% CI) | 2.0 (1.2, 3.3) | 3.3 (2.2, 4.8) | 0.57 (0.30, 1.09); p=0.091 |
| **Time to first CID by type** | | | |
| *CID, % probability of an event to Day 168 (95% CI)* | | | |
| Exacerbation, FEV_1_, SGRQ | 60.2 (56.5, 63.9) | 69.5 (66.1, 72.8) | 0.75 (0.66, 0.85); p<0.001 |
| Exacerbation, FEV_1_, CAT | 60.4 (56.7, 64.1) | 67.1 (63.7, 70.5) | 0.78 (0.69, 0.89); p<0.001 |
| Exacerbation, CAT, SGRQ, TDI^c^ | 69.8 (66.3, 73.2) | 73.4 (70.1, 76.5) | 0.88 (0.78, 0.99); p=0.036 |
| *CID, % probability of an event to Day 168 (95% CI)* | | | |
| FEV_1_ | 30.9 (27.6, 34.6) | 43.3 (39.7, 47.0) | 0.61 (0.52, 0.72); p<0.001 |
| SGRQ | 39.2 (35.7, 43.0) | 41.6 (38.0, 45.4) | 0.88 (0.75, 1.03); p=0.11 |
| CAT | 35.9 (32.5, 39.6) | 36.1 (32.7, 39.7) | 0.95 (0.81, 1.12); p=0.57 |
| TDI | 33.8 (30.4, 37.5) | 35.6 (32.1, 39.3) | 0.96 (0.81, 1.13); p=0.61 |
| Exacerbation | 16.1 (13.6, 19.0) | 19.4 (16.7, 22.4) | 0.80 (0.62, 1.02); p=0.067 |

^a^Symptom severity outcomes presented for Week 24 for SAC-TDI and global assessment of disease severity, for Weeks 21–24 for E-RS, and for Weeks 1–24 for rescue salbutamol use; ^b^data are LS mean (95% CI); ^c^SAC-TDI responders were defined as a ≥1-unit improvement from baseline; ^d^E-RS responders were defined as a reduction of ≥2 from baseline; ^e^overall assessment of change in COPD severity was rated using a seven-point Likert scale (‘Much Better’, ‘Slightly Better’, ‘Better’, ‘No Change’, ‘Slightly Worse’, ‘Worse’, ‘Much Worse’). Ordered response ratios were reported as odds of better response category; ^f^SGRQ responders were defined as a ≥4-point reduction from baseline; ^g^CAT responders were defined as a ≥2-unit improvement from baseline.

CAT, COPD Assessment Test; CI, confidence interval; CID, clinically important deterioration; COPD, chronic obstructive pulmonary disease; E-RS, Evaluating Respiratory Symptoms-COPD; FEV_1_, trough forced expiratory volume in one second; LS, least squares; n, number of responders/patients with an event; N, number of patients with analysable data; SAC-TDI, self-administered computerised Transition Dyspnoea Index; SAL, salmeterol; SGRQ, St George’s Respiratory Questionnaire; UMEC, umeclidinium; VI, vilanterol.
