# Supplementary material for: Efficacy of umeclidinium/vilanterol versus umeclidinium and salmeterol monotherapies in symptomatic patients with COPD not receiving inhaled corticosteroids: the EMAX randomised trial
Source: Respir Res. 2019 Oct 30;20:238. doi: 10.1186/s12931-019-1193-9 (PMC6821007; doi:10.1186/s12931-019-1193-9)
Supplement: Supplementary file 3 — Additional file 3: Table S3. Proportion of responders for symptom severity and health status outcomes – additional timepoints. aSAC-TDI responders were defined as a ≥ 1-unit improvement from baseline; bE-RS responders were defined as a reduction of ≥2 from baseline; coverall assessment of change in COPD severity was rated using a seven-point Likert scale (‘Much Better’, ‘Slightly Better’, ‘Better’, ‘No Change’, ‘Slightly Worse’, ‘Worse’, ‘Much Worse’). Ordered response ratios were reported as odds of better response category; dSGRQ responders were defined as a ≥ 4-point reduction from baseline; eCAT responders were defined as a ≥ 2-unit improvement from baseline. CAT, COPD Assessment Test; CI, confidence interval; COPD, chronic obstructive pulmonary disease; e-diary, electronic diary; E-RS, Evaluating Respiratory Symptoms-COPD; n/N, number of responders/number of patients with analysable data; SAC-TDI, self-administered computerised Transition Dyspnoea Index; SAL, salmeterol; SGRQ, St George’s Respiratory Questionnaire; UMEC, umeclidinium; VI, vilanterol. [file 12931_2019_1193_MOESM3_ESM.docx]

**Additional Table** **3** Proportion of responders for symptom severity and health status outcomes – additional timepoints

|  | **UMEC/VI**  **(N=812)** | **UMEC  (N=804)** | **SAL**  **(N=809)** |
| --- | --- | --- | --- |
| **Symptom severity outcomes** | | | |
| *SAC-TDI focal score* | | | |
| Week 4 |  |  |  |
| SAC-TDI responders^a^, n/N (%) | 410/803 (51) | 342/795 (43) | 333/803 (41) |
| UMEC/VI vs comparator odds ratio (95% CI) | - | **1.40 (1.14, 1.72); p=0.001** | **1.51 (1.23, 1.85); p<0.001** |
| Week 12 |  |  |  |
| SAC-TDI responders^a^, n/N (%) | 414/803 (52) | 331/797 (42) | 340/802 (42) |
| UMEC/VI vs comparator odds ratio (95% CI) | - | **1.53 (1.25, 1.87); p<0.001** | **1.48 (1.21, 1.81); p<0.001** |
| *E-RS total score* | | | |
| Weeks 1–4 |  |  |  |
| E-RS responders^b^, n/N (%) | 236/803 (29) | 202/796 (25) | 188/805 (23) |
| UMEC/VI vs comparator odds ratio (95% CI) | - | **1.26 (1.00, 1.58); p=0.047** | **1.37 (1.09, 1.73); p=0.006** |
| Weeks 9–12 |  |  |  |
| E-RS responders^b^, n/N (%) | 278/802 (35) | 251/793 (32) | 221/800 (28) |
| UMEC/VI vs comparator odds ratio (95% CI) | - | 1.17 (0.95, 1.45); p=0.141 | **1.39 (1.12, 1.72); p=0.003** |
| *Global assessment of disease severity*^c^ | | | |
| Week 4 |  |  |  |
| UMEC/VI vs comparator ordered odds ratio for improvement in response category (95% CI) | - | **1.39 (1.16, 1.67); p<0.001** | **1.42 (1.18, 1.70); p<0.001** |
| Week 12 | | | |
| UMEC/VI vs comparator ordered odds ratio for improvement in response category (95% CI) | - | **1.45 (1.21, 1.75); p<0.001** | **1.50 (1.24, 1.80); p<0.001** |
| **Health status outcomes** | | | |
| *SGRQ total score* | | | |
| Week 4 |  |  |  |
| SGRQ responders^d^, n/N (%) | 354/807 (44) | 301/795 (38) | 310/804 (39) |
| Odds ratio vs UMEC/VI (95% CI) | - | **1.34 (1.09, 1.64); p=0.006** | **1.27 (1.03, 1.56); p=0.022** |
| Week 12 |  |  |  |
| SGRQ responders^d^, n/N (%) | 374/808 (46) | 321/799 (40) | 309/801 (39) |
| UMEC/VI vs comparator odds ratio (95% CI) | - | **1.33 (1.08, 1.63); p=0.006** | **1.41 (1.15, 1.73); p=0.001** |
| *CAT score* | | | |
| Week 4 |  |  |  |
| CAT responders^e^, n/N (%) | 437/800 (55) | 406/794 (51) | 432/803 (54) |
| UMEC/VI vs comparator odds ratio (95% CI) | - | 1.18 (0.96, 1.44); p=0.116 | 1.06 (0.86, 1.29); p=0.588 |
| Week 12 |  |  |  |
| CAT responders^e^, n/N (%) | 456/804 (57) | 411/797 (52) | 419/796 (53) |
| UMEC/VI vs comparator odds ratio (95% CI) | - | **1.26 (1.03, 1.54); p=0.025** | 1.20 (0.98, 1.46); p=0.080 |

^a^SAC-TDI responders were defined as a ≥1-unit improvement from baseline; ^b^E-RS responders were defined as a reduction of ≥2 from baseline; ^c^overall assessment of change in COPD severity was rated using a seven-point Likert scale (‘Much Better’, ‘Slightly Better’, ‘Better’, ‘No Change’, ‘Slightly Worse’, ‘Worse’, ‘Much Worse’). Ordered response ratios were reported as odds of better response category; ^d^SGRQ responders were defined as a ≥4-point reduction from baseline; ^e^CAT responders were defined as a ≥2-unit improvement from baseline.

CAT, COPD Assessment Test; CI, confidence interval; COPD, chronic obstructive pulmonary disease; e-diary, electronic diary; E-RS, Evaluating Respiratory Symptoms-COPD; n/N, number of responders/number of patients with analysable data; SAC-TDI, self-administered computerised Transition Dyspnoea Index; SAL, salmeterol; SGRQ, St George’s Respiratory Questionnaire; UMEC, umeclidinium; VI, vilanterol.
