# Supplementary material for: Efficacy of umeclidinium/vilanterol versus umeclidinium and salmeterol monotherapies in symptomatic patients with COPD not receiving inhaled corticosteroids: the EMAX randomised trial
Source: Respir Res. 2019 Oct 30;20:238. doi: 10.1186/s12931-019-1193-9 (PMC6821007; doi:10.1186/s12931-019-1193-9)
Supplement: Supplementary file 4 — Additional file 4: Table S4. LS mean change from baseline and proportion of respondersa for E-RS subdomains at Weeks 21–24. aE-RS responders were defined as a reduction of ≥1 unit from baseline for E-RS breathlessness score, and a reduction of ≥0.7 units from baseline for cough and sputum, and chest scores. CI, confidence interval; CFB, change from baseline; E-RS, Evaluating Respiratory Symptoms-COPD; LS, least squares; n/N, number of responders/number of patients with analysable data; SAL, salmeterol; UMEC, umeclidinium; VI, vilanterol. [file 12931_2019_1193_MOESM4_ESM.docx]

**Additional Table 4** LS mean change from baseline and proportion of responders^a^ for E-RS subdomains
at Weeks 21–24

|  | **UMEC/VI**  **(N=812)** | **UMEC  (N=804)** | **SAL  (N=809)** |
| --- | --- | --- | --- |
| *E-RS breathlessness score at Weeks 21–24* |  |  |  |
| LS mean CFB (95% CI) | -0.67 (-0.83, -0.52) | -0.40 (-0.56, -0.24) | -0.22 (-0.38, -0.06) |
| UMEC/VI vs comparator mean difference (95% CI) | - | **-0.27 (-0.50, -0.05)**  **p=0.016** | **-0.46 (-0.68, -0.23)**  **p<0.001** |
| E-RS breathlessness score responders, n/N (%) | 253/809 (31) | 208/800 (26) | 195/808 (24) |
| UMEC/VI vs comparator odds ratio (95% CI) | - | **1.32 (1.06, 1.64)**  **p=0.015** | **1.43 (1.14, 1.78)**  **p=0.002** |
| *E-RS cough and sputum score at Weeks 21–24* |  |  |  |
| LS mean CFB (95% CI) | -0.45 (-0.53, -0.36) | -0.38 (-0.46, -0.29) | -0.32 (-0.41, -0.23) |
| UMEC/VI vs comparator mean difference (95% CI) | - | -0.07 (-0.20, 0.05)  p=0.247 | **-0.13 (-0.25, -0.00)**  **p=0.042** |
| E-RS cough and sputum score responders, n/N (%) | 263/809 (33) | 218/800 (27) | 218/808 (27) |
| UMEC/VI vs comparator odds ratio (95% CI) | - | **1.27 (1.01, 1.58)**  **p=0.038** | **1.27 (1.01, 1.58)**  **p=0.037** |
| *E-RS chest score at Weeks 21–24* |  |  |  |
| LS mean CFB (95% CI) | -0.39 (-0.48, -0.29) | -0.22 (-0.31, -0.12) | -0.15 (-0.25, -0.05) |
| UMEC/VI vs comparator mean difference (95% CI) | - | **-0.17 (-0.31, -0.04)**  **p=0.014** | **-0.24 (-0.37, -0.10)**  **p<0.001** |
| E-RS chest score responders, n/N (%) | 238/809 (29) | 188/800 (24) | 192/808 (24) |
| UMEC/VI vs comparator odds ratio (95% CI) | - | **1.43 (1.14, 1.80)**  **p=0.002** | **1.36 (1.08, 1.71)**  **p=0.008** |

#### ^a^E-RS responders were defined as a reduction of ≥1 unit from baseline for E-RS breathlessness score, and a reduction of ≥0.7 units from baseline for cough and sputum, and chest scores.

CI, confidence interval; CFB, change from baseline; E-RS, Evaluating Respiratory Symptoms-COPD; LS, least squares; n/N, number of responders/number of patients with analysable data; SAL, salmeterol; UMEC, umeclidinium; VI, vilanterol.
