# Supplementary material for: Efficacy of umeclidinium/vilanterol versus umeclidinium and salmeterol monotherapies in symptomatic patients with COPD not receiving inhaled corticosteroids: the EMAX randomised trial
Source: Respir Res. 2019 Oct 30;20:238. doi: 10.1186/s12931-019-1193-9 (PMC6821007; doi:10.1186/s12931-019-1193-9)
Supplement: Supplementary file 5 — Additional file 5: Table S5. List of investigators. [file 12931_2019_1193_MOESM5_ESM.docx]

**Additional Table 4** List of investigators

| **Name (Surname, First name)** | **Name (Surname, First name)** | **Name (Surname, First name)** |
| --- | --- | --- |
| Abboy, Chandar  Abrahams, Roger  Alhakim, Mazin A.A.F.  Aliani, Maria  Anees, Syed  Aramayo, Norma  Arce, German  Arelin, Katrin  Baker, David  Ballenberger, Sabine  Banerjee, Anup  Barro, Analia  Beck, Ekkehard  Benedix, Andreas  Berg, Peter  Bergna, Miguel  Berndtsson Blom, Katarina  Bernstein, David  Bloch, Mark  Bocca Ruiz, Pedro Xavier  Bollmann, Lutz  Boscia III, Joseph  Botnick, Warren  Bouda, David  Breedt, Johannes  Brunner, Falk  Califano, Mariano  Cambursano, Victor  Carswell, James  Catapano, Giosuè  Chandran, Ravi  Chevts, Julia  Chirino Navarta, Alejandro  Chung, Steven  Contzen, Christel  Cruz, Humberto  Custers, Frank L.J.  Cuttitta, Giuseppina  Dabove, Fabian  D'Amato, Maria  De La Cruz, Luis  de Roux, Andrés  De Salvo, María  De Teresa Parreño, Luis  Deckelmann, Regina  Deimling, Andreas  Del Donno, Mario  Denenberg, Michael  Dunn, Leonard  Dzongowski, Peter  Eckermann, Tamara  Eckhardt, Gerald  Ehlers, Martin  Eich, Andreas  Einenkel, Andreas  Elías, Pedro  Eliasson, Ken  Ellis, Christopher  Erb, David  Ern, Guido  Esselmann, Albert  Feldman, Gregory  Ferguson, Murdo  Fernández, Marcelo  Figueroa, Sandra  Fischer, Rainal Foerster, Karin Fogarty, Charles Forster, Andreas  Fourie, Nyda  Fulat, Muhammed Gessner, Christian  Giovini, Vanina | Ginko, Thomas  Girodet, Pierre-Olivier  Glas, Wiecher O.  Goosens, Martijn  Graif, Joseph  Greenspoon, Todd  Grigat, Christine  Grullon, Kevin  Guenzel, Andrea  Gupta, Anil  Hammerl, Peter  Heinz, Gerd-Ulrich  Hellgren, Margareta Hernández-Colín, Dante Daniel Hoeffgen, Marc Hoek, Boudewijn A. Hoffmann, Martin Hoheisel, Gerhard Hoosen, Farzana Hyers, Thomas Ignacio Garcia, Jose Maria Kaelin, Jr., Thomas Kaye, Mitchell Kehm, Stefan Keller, Claus Kirschner, Joachim Kleinecke-Pohl, Uwe Korenblat, Phillip Koskinen, Pekka Labelle, Andrew Lachance, Pierre Landry, Daniel Larivey, Virginia Larrateguy, Luis Lawriwskyj, Verónica Lazzari Agli, Luigi Arcangelo Lienert, Thomas Lillo, Joseph Lindberg, Anne Linnhoff, Anneliese Ludwig-Sengpiel, Andrea Luts, Anders  Luttermann, Matthias Lynn, Lon Malamud, Patricia Mannarino, Silvina Mansour, Abbas Marten, Irmgard Martínez Navarro, Encarnación Martínez Piera, Pyrene Massola, Fernando Mateos Caballero, Luis Mattarucco, Walter Mazza, Francesco Mazza, Giuseppe McGuire, Michael Menzella, Francesco Mincheva, Roxana Mitha, Essack Mohan, Gowdhami Molina, Alicia Mookadam, Mohamed Mosl, Beate Muzi, Giacomo Nischik, Ruth  Olsson, Åke O'Mahony, John Overlack, Axel  Patel, Nancy Pegliasco, Hervé  Pek, Bonavuth Peldschus, Meike Pigearias, Bernard | Pont Barrio, Francesc  Pritchett, Kevin  Promencio, Federico  Pudi, Krishna  Rañó Pérez, Ana  Raso, Ernesto  Rein-Hedin, Erik  Rey, Luisa  Richter, Daphne  Robinette, Emory  Rodríguez Moncalvo, Juan  Rodriguez, Alicia  Roesler, Stefanie  Rojas, Ramón Roldán Sánchez, Juan  Romberg, Kerstin  Sabato, Eugenio  Saez Scherbovsky, Pablo  Sanduzzi Zamparelli, Alessandro  Saueressig, Heiner  Schaefer, Axel  Schaefer, Christian  Schaper, Lennart  Schenkenberger, Isabelle  Schlegel, Volker  Schlenska, Christian  Schmidt, Olaf  Schuermann, Wolfgang  Shealy, Keith  Sigal, Barry  Siler, Thomas  Smith, David  Sosso, Adriana  Spanevello, Antonio  Spangenthal, Selwyn  St-Amour, Eric  Steinmetz, Karl Otto  Stolpe, Christoph Surdulescu, Sever  Tayob, Mohammed Siddique  Tellier, Guy  Tolcachier, Alberto  Ullman, Anders  van der Sar, Simone  van Zyl, Lelanie  van Zyl-Smit, Richard  Vandeleur, James  Vatrella, Alessandro  Verdier, Serge  Verra, Fernando  Victorio, Carlos  Vismane, Liana  Volgmann, Lutz  Voloshyna, Olga  von Engelhardt, Charlotte  von Muenchhausen, Candy  Vries, Peter M.J.M. de  Walle, Viviënne E.K.M. van de  Wayne, Jeffrey  Wehbe, Luis Westphal, Arite  Wiederhold, Conrad  Wiemer, Silke  Wilhase, Agatha Wilhelms, Daniel  Winkels, Wilhelm  Winkler, Joerg  Wright, Patrick Wustmann, Thomas  Zachgo, Wolfgang  Zielen, Stefan Zuriqat, Muqdad |
